# Sustainable Pet Diets: A Leading Effective Altruism Issue

**DOI:** 10.3390/ani16030460

**Published:** 2026-02-01

**Authors:** Andrew Knight

**Affiliations:** 1 School of Environment and Science, Griffith University, Nathan, QLD 4111, Australia; a.knight@griffith.edu.au; 2 School of Veterinary Medicine, College of Environmental and Life Sciences, Murdoch University, Murdoch, WA 6150, Australia; 3 Animal Welfare Research Group, Faculty of Health and Wellbeing, University of Winchester, Winchester SO22 4NR, UK; 4 Sustainable Pet Food Foundation, London E4 6AG, UK

**Keywords:** cat, dog, effective altruism, environment, ethics, pet food, pet health, sustainability

## Abstract

While animal-derived ingredients continue to dominate pet food, mounting animal welfare and environmental pressures are starting to reshape the market—opening the door to plant-based and cultivated meat alternatives for dogs and cats. This study assessed the effective altruism case for more sustainable pet food options, using the scale, neglectedness, and tractability framework, and found strong alignment across all three dimensions. By 2018, at least 9% of farmed land animals were fed to companion dogs and cats globally, with more consumed by average dogs (13) than by average people (9) annually. A global transition to nutritionally sound vegan pet diets could spare seven billion farmed land animals and many billions of marine animals from slaughter and could feed 519 million additional people using food energy savings. Such a transition for dogs alone could eliminate 1.5 times the quantity of greenhouse gases produced annually by the UK and free up land larger than Mexico. Yet, sustainable pet food is a highly neglected issue in terms of funding, time, and talent. The issue appears tractable; 13–18% of dog and cat guardians would consider vegan pet diets if their concerns about them were addressed. Assuming only one dog or cat per guardian, at least 70 million dogs and 86 million cats worldwide could potentially be transitioned to vegan diets, with the true figures probably several times higher. Sustainable pet diets, therefore, represent a highly impactful yet overlooked opportunity to reduce farmed animal consumption, mitigate associated environmental impacts, and improve food security.

## Simple Summary

Animal-based ingredients dominate dog and cat food, but growing concerns about animal welfare and the environment are increasing interest in more sustainable options, such as plant-based and cultivated meat-based pet foods. This study evaluated the case for sustainable pet food using the effective altruism framework of scale, neglectedness, and tractability, and found strong support on all three. Globally, by 2018, at least 9% of land animals farmed each year were used to feed dogs and cats, with more consumed by average dogs (13) than by average people (9) annually. Switching to nutritionally sound vegan pet diets worldwide could spare around seven billion land animals and many billions of marine animals from slaughter, while freeing enough food energy to feed 519 million people. Transitioning only dogs could cut greenhouse gas emissions equal to 1.5 times the UK’s annual output and free land larger than Mexico. Despite these benefits, sustainable pet food receives little funding, attention, or talent. However, the issue is tractable: 13–18% of pet guardians would consider vegan diets if their concerns were met. Assuming only one dog or cat per guardian this could allow at least 70 million dogs and 86 million cats to transition globally, with the true figures probably several times greater. Overall, sustainable pet diets are a largely overlooked but high-impact opportunity to reduce farmed animal consumption, lower environmental harm, and improve food security.

## 1. Introduction

### 1.1. Effective Altruism

The term ‘effective altruism’ (EA) originated when Oxford University scholars William MacAskill and Toby Ord founded the Center for Effective Altruism in 2011. EA is a philosophy and social movement focused on identifying the most effective ways to help others. It uses evidence and reason to guide decisions about how money, time, and resources should be spent to maximize positive impact [1,2]. Issues are prioritized based on how large in scale, how neglected, and how tractable (solvable) they are. EA decision-making emphasizes impartiality, meaning that no extra weight is given to interventions simply because they benefit individuals who are more similar to, or geographically closer to, the decision-makers [1].

EA has gained global recognition, with its principles being employed by tens of thousands of people in over 70 countries [1]. With an estimated 10 million nonprofit organizations globally [3], donors can easily feel conflicted about where to allocate their resources. Moreover, charities can differ by orders of magnitude in how many individuals they are able to help with a set amount of resources [1]. EA provides a valuable framework for navigating these difficult decisions and maximizing the effectiveness of charitable giving. EA prioritizing has often considered human diets, but to date, has had very little focus on companion animal diets.

### 1.2. Sustainable Pet Food

In the following, sustainable dog or cat food is defined as that which is vegan (fully plant-based or microbial protein-based) or based on cellular agriculture. Cellular agriculture products fall into two categories: *cell-based*, such as cultivated meat and other foods made from animal cells grown in culture; and *acellular*, such as recombinant animal proteins and other ingredients produced through microbial fermentation in safe microbial cell factories using genetic engineering or synthetic biology [4,5].

Until recently, commercially available diets of these kinds, carefully designed and manufactured to be nutritionally sound, along with studies of their health effects on dogs and cats, were few. This may largely explain the lack of focus on sustainable pet diets by the EA movement. By 2025, however, fully plant-based pet foods were relatively widely available—often from online vendors [6], and diets based on microbial protein or cultivated meat were rapidly developing. In February 2025, the UK-based company Meatly’s ‘Chick Bites’ for dogs became the world’s first cultivated meat-based pet food product to be sold commercially [7]. In the same month, a nutritionally complete dog food was released that contained ‘FeedKind’, a first-of-its-kind microbial protein produced by culturing the harmless bacterium Methylococcus capsulatus [8].

There is growing interest in sustainable diets for dogs and cats. Globally, the vegan pet food market was valued at USD 10 billion in 2020 [9] and USD 27 billion in 2024 [10] and is forecast to more than double in value to USD 57 billion by 2034—reportedly much faster than the conventional pet food market [11]. Several key drivers are fueling this trend. Consumers are increasingly concerned about the welfare of farmed animals, which can lead to feelings of conflict associated with feeding meat to companion animals—often referred to as the ‘vegetarian’s dilemma’ or the ‘animal lovers’ paradox’ [12,13]. Additionally, an awareness of the environmental impacts of animal agriculture is prompting some dog and cat guardians to seek out more sustainable pet food options [14]. (In this article, the term ‘guardian’ rather than ‘owner’ is used, partly because some companion dogs and cats are stray or colony animals, community-owned or housed in animal shelters, and lack specific owners). There is also a growing body of evidence to suggest that dogs and cats maintained on well-formulated vegan diets experience equivalent or superior health outcomes compared to those fed conventional animal-based diets [14].

This study aimed to assess the EA case for increasing consumer demand for more sustainable dog and cat food options, based on the criteria of scale, neglectedness, and tractability. These more sustainable dog and cat food options were considered to include vegan (fully plant-based or microbial protein-based), vegetarian (which may include animal products other than meat, such as eggs or dairy products), plant-based (i.e., containing a greater proportion of plant-derived ingredients than traditional formulations), or cultivated meat-based options. Insect-based pet food was excluded due to concerns regarding the environmental sustainability of insect farming [15]. Furthermore, a growing body of evidence suggests that insects may be capable of feeling pain [16], raising considerable ethical concerns about their intensive farming for use within the food system. The latter concern is accentuated by their size; insects are considerably smaller than conventionally farmed animals and, thus, would need to be farmed in vastly greater numbers to meet demand.

## 2. Scale

There is a very large market for dog and cat food, as the global pet food market was valued at USD 132.4 billion in 2025 [17], and dog and cat food accounted for 95% of sales [18]. This reflects the high prevalence of dog and cat guardianship; more than half of global households care for a dog or cat, rising to two-thirds in the US [18,19]. In 2024, there were an estimated 528 million companion dogs and 476 million companion cats worldwide, with these figures expected to increase alongside human population growth and continued development in many nations [20,21]. Correspondingly, the global pet food market is projected to nearly double to USD 247.7 billion USD by 2035 [17]. Given that animal-derived ingredients comprise an estimated 53% of all dog and cat food [22], this growth poses significant animal welfare, ethical, and environmental concerns.

### 2.1. Animal Welfare and Ethical Implications

Animals used in the production of pet food commonly experience welfare challenges throughout their lives; an estimated 94% of all farmed land animals and farmed fish combined live in intensive farms [23]. They endure a range of adverse conditions, including overcrowding, involuntary confinement in relatively barren environments—frequently with minimal opportunities to exercise highly motivated natural behaviors, risks of disease and injury, and bodily mutilations without anesthetics or analgesics. Additionally, these welfare challenges are often exacerbated by selective breeding to maximize productivity. These animals are usually slaughtered at a very premature stage compared to their natural lifespans, and some experience ineffective stunning and/or painful slaughter methods [24].

A vast number of sentient animals are negatively affected by the production of animal-based pet food. Globally, 99% of all animals used by humans are farmed animals [25], with a significant proportion fed to companion animals. After accounting for the use of animal byproducts (ABPs)—which are the parts of animals not normally consumed by humans, within pet food, 8.9% of all terrestrial farmed animals globally were consumed by dogs (7.7%) and cats (1.2%) by 2018 [22]. Transitioning all companion dogs and cats to nutritionally sound vegan diets could therefore spare 7.0 billion land animals annually (6.0 billion for dogs, 0.9 billion for cats), in addition to many billions of marine animals. In the US alone, where at least 20% of terrestrial farmed animals were consumed by dogs and cats by 2020, such a shift could save 2.0 billion terrestrial animals each year, plus billions of marine animals [22]. These data were previously calculated [22] and are reproduced within Table 1 and Table 2.

These calculations from Knight [22] were then extended within the current study to determine the number of land animals spared from slaughter per average dog or cat transitioned onto a nutritionally sound vegan diet, both annually, and if such a diet was followed for an average dog or cat lifespan (Table 1 and Table 2). Human data is provided for comparison.

These data were calculated by considering both the numbers of dogs, cats, and humans within the US in 2020, and globally in 2018, as provided by Knight [22], and the average life expectancies within these populations. As explained in Knight [22], US data for 2020 were used. However, reliable global dog and cat population numbers for 2020 were unavailable, so 2018 figures were used for the global calculations. These populations comprised (in millions): US (2020): humans—329.0, dogs—86.3, cats—61.1; globally (2018) humans—7684, dogs—471, cats—373.

For dogs and cats, life expectancy varies considerably depending on breed size, health status, sex, and other factors, but averages are nevertheless possible to determine. Analyzing records of 13,292,929 dogs and 2,390,078 cats sourced from over 1000 US veterinary clinics, Montoya et al. [26] determined that average life expectancies were 12.69 years for dogs and 11.18 years for cats. For humans in the same year (2023), life expectancy was 78.4 years in the US [27] and 73.4 years globally [28].

These average life expectancies were combined with population numbers to create the new data provided in Table 1 and Table 2. Within the US in 2020, the number of farmed land animals consumed to feed dogs was, on average, 20.02/year and 254.03 over an average lifespan. For cats, these numbers were 3.67 and 41.08. Globally in 2018, the equivalent numbers were: dogs: 12.79/year and 162.33 over an average lifespan; and for cats: 2.52 and 28.14. It is noteworthy that, globally, by 2018 fewer farmed land animals were consumed within the diets of ordinary people (9.28) than average dogs (12.79) each year.

There is a considerable disparity between US and global consumption of farmed animals, probably reflecting a higher proportion of animal-based ingredients (which are costlier than plant-based ingredients) fed to pets in higher-income nations like the US. Similar disparities are also evident for human consumption levels.

Nevertheless, both within the US and globally, it is clear that tens to hundreds of farmed land animals are killed to support the lifetime consumption of both dogs and cats fed meat-based diets. These animals would be spared from slaughter through the use of nutritionally sound vegan diets, as well as billions of fish and marine animals when total populations are considered [22].

My prior study [22] also quantified the number of additional humans who could be fed using the food energy savings associated with a global transition of companion animals to vegan diets: 449.1 million people if companion dogs transitioned, and 69.7 million people if companion cats transitioned. These figures exceeded the 2018 populations of the entire European Union and the UK, respectively, together amounting to 6.8% of the total human population. Alternatively, the same food energy savings could be used to feed 749.9 million additional dogs (~160% of the 2018 global companion dog population of 471 million) if companion dogs transitioned, and 705.6 million cats (~190% of the 2018 global companion cat population of 373 million) if companion cats transitioned. These food energy savings demonstrate the potential for vegan pet diets to contribute to global food security, provided these savings are effectively redirected.

All of these figures are likely underestimates; conservative assumptions were made regarding global companion animal populations and their energy requirements, and the calculations did not account for the millions of working dogs, animal shelter pet populations, or human-fed stray or free-roaming dogs and cats. Furthermore, slaughter statistics and pet guardianship rates from 2018 were used, both of which have since increased substantially.

The production of animal-based pet food is, thus, an issue that clearly meets the EA criterion of scale, both in terms of the number of affected individuals and the degree to which they are negatively affected, making this a particularly high-impact cause area.

### 2.2. Environmental Implications

There are also substantial environmental costs associated with the production of conventional animal-based pet food. Animal agriculture is a leading driver of climate change and biodiversity loss, accounting for an estimated 20% of global greenhouse gas (GHG) emissions annually and over one-third of all habitable land use [29,30]. An analysis by Okin [31] revealed that companion animal diets were responsible for 25–30% of the environmental impacts of animal agriculture in the US. Similarly, a study of Japanese consumption found that the dietary environmental footprint of one Japanese person was less than the dietary environmental ‘paw print’ of one medium-sized dog [32].

In addition to the animal welfare and food security benefits, my aforementioned study [22] calculated the environmental impacts of transitioning all companion dogs and cats globally onto nutritionally sound vegan diets. Such a transition could free up land areas larger than Mexico (dogs) or Germany (cats), reduce GHG emissions by amounts greater than all those emitted by the UK (dogs) or New Zealand (cats), and save freshwater volumes exceeding all renewable freshwater in Denmark (dogs) or Jordan (cats). As discussed in Section 2.1 ‘Animal welfare and ethical implications’, these estimates are conservative, and the true benefits are probably considerably higher. For example, such a global transition for pet dogs would actually save GHG emissions equal to 1.5 times those emitted annually by the UK [33].

Consistent with these findings, a recent life cycle assessment of 31 commercially available dry dog foods in the UK—comprising vegan and vegetarian, red meat-based (beef or lamb), poultry-based, and veterinary renal (kidney disease-focused) diets—found that the vegan and vegetarian foods had the lowest impacts across all environmental indicators [34]. Beef-based foods had the greatest impacts, with intermediate impacts attributable to the poultry-based and veterinary renal diets. The GHG emissions associated with the beef-based foods were over ten times greater than those of the vegan and vegetarian formulas (31.47 kg vs. 2.82 kg CO_2_-eq per 1000 kcal). Consequently, over the typical nine-year lifespan of an average sized-dog such as a 20 kg Labrador, a vegan or vegetarian diet would result in GHG emissions equivalent to 2.8 return flights from London to New York, compared to 31.3 such flights for a beef-based diet. This represents a total saving of 28.5 flights, or 3.2 flights per average year of canine life. Similarly, relative to the beef-based foods, the vegan and vegetarian foods had substantially lower land use (2.73 m^2^ vs. 102.15 m^2^ per 1000 kcal) and water use (249 L vs. 575 L freshwater per 1000 kcal). Beef-based diets were also associated with 14.3 times more acidifying emissions and 16.4 times more eutrophying emissions than the vegan and vegetarian alternatives [34].

Cultivated meat-based and microbial protein-derived pet foods are currently less developed than vegan pet foods, and hence, environmental data regarding their production is very limited. Nevertheless, preliminary research suggests these technologies could offer substantial environmental benefits. Biotechnology company ‘Calysta’ (San Mateo, CA, US), produces the ‘FeedKind’ microbial protein via gas-based fermentation, thus eliminating the need for agricultural land. Calysta’s plant can generate 100,000 metric tons of protein per year using just 0.1 km^2^ of developed land [35]. Producing the same amount of protein as beef would require 44,640 km^2^—446,400 times more land [36]. Moreover, replacing soy protein with FeedKind could save approximately nine million cubic meters of water, equivalent to 3600 Olympic swimming pools [35]. An analysis commissioned by cultivated pet food manufacturer BioCraft (Vienna, Austria) found that the production of their cultivated meat emits only 8.3% of the GHG emissions associated with beef-based derivatives in conventional pet food (1.73 kg vs. 21.28 kg CO_2_ per kg) [37].

It is often assumed that ABPs would be discarded if not used in pet food, and that their use therefore reduces or even offsets the pet food industry’s environmental footprint [31,38,39,40]. However, this is a misconception; multiple sectors compete for ABPs due to their diverse applications, including in livestock feed, fertilizers, pharmaceuticals, and renewable energy feedstocks. The pet food industry only uses around 25% of all ABPs [22]. Indeed, growing demand for ABPs from the renewable energy sector has raised concerns about potential companion animal food shortages if alternative protein sources are not prioritized [41]. The use of ABPs in pet food simply increases demand for them. 

Furthermore, ABPs constitute 31–39% of an average livestock animal carcass used in dog or cat food, while human-grade components such as meat comprise 53–59%. This means that more carcasses are required to produce any given quantity of animal-sourced ingredients when ABPs are used, compared to the use of human-grade components: 1.4 times as many carcasses for dog food, and 1.9 times as many for cat food [22]. The use of ABPs instead of human-grade components therefore increases the number of farmed animal carcasses used during pet food production, exacerbating the associated environmental (and ethical) concerns.

## 3. Neglectedness

Despite the scale of the ethical and environmental problems associated with producing animal-based pet food [22], efforts to promote a transition toward more sustainable alternatives are highly neglected, even within animal advocacy and environmental circles. To the author’s knowledge, only one organization—the UK-based Sustainable Pet Food Foundation (SPFF)—focuses specifically on sustainable pet food research and advocacy, and only a tiny number (~two by late 2025) of animal and environmental advocates are known to work on this issue full-time. This compares to hundreds of organizations globally, collectively employing thousands of full-time staff, that work on human dietary change, within the animal advocacy and environmental movements.

Furthermore, although there are over 40 (and growing) companies globally producing vegan or cultivated meat-based pet food [6], their primary focus is on creating and marketing specific products, rather than conducting broader initiatives aimed at accelerating a transition toward more sustainable pet foods. These companies’ marketing budgets are also extremely modest compared to those of conventional pet food companies.

Interventions aimed at promoting a transition toward more sustainable pet foods have received minimal funding relative to their potential impact on farmed animal welfare. In 2020, the farmed animal advocacy community’s total budget was around USD 220 million [42], which had risen to USD 260 million by 2024 [43,44]. Funding for vegan pet food research and advocacy is believed to be very far below USD 1 million annually, which is well below 0.5% of the farmed animal advocacy movement’s budget. A December 2025 search of transparent EA-aligned animal welfare grant databases (Animal Charity Evaluators, Open Philanthropy, and EA Grants) using terms including ‘companion animal’, ‘pet food’, ‘dog’, and ‘cat’, revealed no relevant grants covering this area.

## 4. Tractability

Tractability within this field largely depends on the willingness of guardians to transition their companion animals to sustainable diets. Three large-scale (1000+ respondents), peer-reviewed studies have assessed consumer attitudes towards more sustainable pet diets, all indicating that a significant proportion of guardians are open to such diets [13,45,46]. Dodd et al. [13] surveyed 3673 dog and/or cat guardians primarily from North America, Europe, and Oceania. Although only 1.6% of dogs and 0.7% of cats were fed a vegan diet, 35% of respondents not currently feeding their companion animals such a diet were interested in doing so. Additionally, some colleagues and I recently published two studies regarding consumer acceptance of more sustainable dog [45] and cat [46] diets. In our dog diet study, 84.2% (2188/2596) of guardians reported feeding their dogs meat-based diets. Among this group of respondents who answered, 24.4%, 16.6%, 13.4% and 7.1% considered cultivated meat-based, vegetarian, vegan, and fungi-based diets acceptable, respectively [45]. In our cat diet study, 88.8% (1226/1380) of guardians surveyed reported feeding their cats meat-based diets. Among this group of respondents who answered, the acceptability of alternative diets was 33.1% for cultivated meat-based, 18.2% for vegan, 13.8% for vegetarian, and 10.4% for fungi-based diets [46]. Participants were able to select multiple responses.

Based on these results, at least 70 million dogs and 86 million cats worldwide could theoretically be transitioned to vegan diets, if guardians’ concerns about such diets could be met. These figures were calculated by first determining the global numbers of companion dogs and cats not currently fed vegan diets, by multiplying the 2024 global populations of companion dogs (528 million) and cats (476 million) by the percentages not currently fed vegan diets—98.4% and 99.3%, respectively [13]. For this step, the percentages from Dodd et al. [13] were used rather than those from our studies [45,46], because our studies deliberately targeted guardians more likely to feed vegan diets, to facilitate concurrent studies of health effects [47,48]. Hence, the Dodd et al. [13] figures more accurately predict the interest of average pet guardians in vegan diets.

The resulting global numbers of companion dogs and cats not currently fed vegan diets (520 million and 473 million, respectively) were then multiplied by the percentages of guardians open to vegan pet diets (13.4% for dogs [45] and 18.2% for cats [46]). For these calculations, our larger, more recent studies were used [45,46], as these indicated the acceptance of vegan diets by those currently feeding meat-based diets. Collectively, these results indicate that over 150 million companion dogs (70 million) and cats (86 million) could be transitioned onto vegan diets.

However, the true numbers are probably several multiples of these, as many dog and cat guardians have more than one of each animal, which is simplistically assumed above. Additionally, conservative percentages of guardians open to vegan diets for dogs (13.4%) and cats (18.2%) were used, which were far lower than the 35% for dogs and cats collectively reported by Dodd et al. [13]. Additionally, awareness of vegan pet diets is likely to be much higher today than when these surveys were conducted (before 2019 for Dodd et al. [13], and in 2020 for Mace et al. [45,46]).

### 4.1. Theory of Change

**Figure 1:**
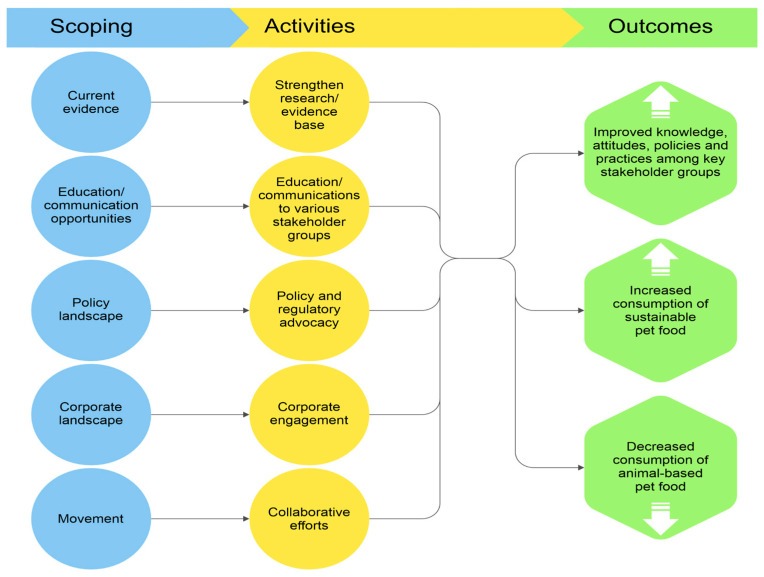
The Sustainable Pet Food Foundation’s theory of change for increasing the consumption of sustainable pet food. Source: SPFF.

To realize the potential provided by a tractable issue, a plausible Theory of Change (ToC) is required. The significant potential for benefits associated with a widespread transition towards more sustainable pet diets could be realized via the pathway outlined in Figure 1. This depicts an example of a ToC for increasing the consumption of sustainable pet food and decreasing the consumption of animal-based pet food, provided by the SPFF. The ToC posits that this can be achieved through five key workstreams: research, education and communication, policy and regulatory advocacy, corporate engagement, and collaborative efforts. These are discussed in the following:

The research workstream includes undertaking studies on sustainable pet food and related topics, peer-reviewing relevant research, and engaging with other researchers. These activities could be expected to lead to outputs such as publications in reputable scientific journals, scientific and industry conference presentations, and contributions that support the work of other researchers. In turn, these outputs should increase both the quantity and quality of research on sustainable pet food, attract more researchers to the field, and ultimately strengthen the overall evidence base exploring sustainable pet diets.

The second workstream focuses on education and communication. It involves creating and disseminating key summaries, articles, infographics, videos, and other educational materials focused on sustainable pet food and related research, as well as issuing press releases and participating in interviews. It also includes targeted communication campaigns for specific stakeholder groups—for example, consumer-focused campaigns that address key concerns about sustainable pet diets (see Section 4.2 ‘Consumer concerns’). These activities can be expected to result in a wide range of communication outputs published across journals, websites, and social media platforms, along with associated media coverage. These outputs should ensure that online searches for sustainable pet food (and related terms) increasingly return high-quality communication materials, which should improve awareness, knowledge, and attitudes about sustainable pet diets among key stakeholders.

The policy and advocacy workstream involves researching existing regulations, policies, and industry practices related to pet food; developing policy recommendations, briefs, and reports for regulators, legislators, and other key policy stakeholders; and devising outreach strategies to inform and influence these audiences. These activities should result in the publication of a range of policy materials and direct engagements with relevant stakeholders. Such outputs ought to increase awareness among decision-makers about the benefits of sustainable pet diets, contribute to improved policies and actions (such as updated regulations or adoption of recommendations), and support greater funding for research, development, and communication focused on sustainable pet food.

The corporate engagement workstream focuses on mapping the funding, investments, policies, and affiliations of mainstream pet food manufacturers, retailers, and related organizations; conducting presentations at pet food conferences; and directly engaging manufacturers and trade associations through meetings and other outreach efforts. The outputs of these activities would include published reports, rankings, insights, and recommendations for the pet care industry, manufacturers, and consumers, as well as productive engagements with manufacturers and pet care industry professionals. One example of such engagement is discussions with retailers about optimal labeling practices, as labels and packaging appear to be the most influential information sources shaping dog and cat guardians’ pet food choices [45,46]. Collectively, these outputs are expected to drive greater investment and innovation in sustainable pet diets; expand the availability, variety, and affordability of sustainable pet food options; and increase the sales and market share of sustainable pet foods.

The final workstream within the SPFF’s ToC focuses on collaborative efforts. Key activities include engaging, educating, and inspiring the animal advocacy, pet guardian and environmental communities; supporting complementary initiatives that aim to reduce the use of farmed animal products in pet foods; and fostering collaboration among animal shelters, manufacturers, and other key stakeholders. These activities are expected to lead to productive engagements across communities with shared interests, as well as the dissemination of communication materials and educational messages through these networks.

The anticipated outcomes of these workstreams include improved knowledge, attitudes, policies, and practices regarding sustainable pet diets within these communities; greater normalization and mainstream acceptance of sustainable pet diets; and increased demand for sustainable pet food within these communities.

The SPFF’s ToC hinges on certain key assumptions, including that demand for sustainable pet food will replace demand for conventional animal-based pet food. Some research has suggested that vegan human foods are consumed in addition to, rather than in place of, animal products. Cuffey et al. [49], for example, found that household spending on meat did not decrease in the month following the first purchase of a plant-based meat alternative (although it dropped by 75% in the following months), and Trewern et al. [50] found that the Veganuary campaign (which encourages consumers to trial vegan diets every January) resulted in increased plant-based purchasing, without displacement of meat sales.

However, a broader review of the literature on this topic by Bryant Research [51] concluded that the consumption of animal product alternatives is significantly negatively correlated with the consumption of animal products within the same category (i.e., meat or dairy) [52]. Similarly, Slade [53] found that a one-gallon increase in plant-based milk sales corresponded to a 0.43–0.60-gallon reduction in dairy milk sales. Crucially, pet diets are considerably less varied than human diets, with guardians tending to feed one main food source to their companion animals for extended periods of time. Common human concerns, such as declining or variable motivation to maintain a new diet, are significantly less relevant for pet diets. Thus, the replacement effects observed when alternative proteins are used within pet diets are likely to be significantly greater than those occurring within human diets.

The SPFF’s ToC also assumes that dissemination efforts will reach guardians who are open to changing their companion animals’ diets. This is considered a reasonable assumption. Existing research articles about the benefits of sustainable pet food have largely been published open-access, sometimes receiving hundreds of media mentions globally (e.g., [21,22]), and generating substantial interest in relevant social media fora.

Figure 1 further theorizes that the research conducted addresses the main concerns or misconceptions that currently hinder broader adoption of sustainable pet diets. This is deemed a valid assumption, as guardians’ main concerns are well understood; across the three aforementioned large-scale studies assessing consumer attitudes towards more sustainable pet diets, guardians most frequently cited concerns regarding health outcomes, nutritional soundness, quality, palatability, availability, and veterinary approval [13,45,46]. The following section examines the validity of these concerns, which have in recent years been the focus of a significant body of research.

### 4.2. Consumer Concerns

#### 4.2.1. Health Outcomes

There is growing evidence to suggest that nutritionally sound vegan or vegetarian pet diets result in equivalent or superior health outcomes compared to meat-based diets. By the start of 2026, 12 studies had assessed health outcomes in dogs maintained on vegan or vegetarian diets. Eleven of these studies demonstrated equivalent or superior health outcomes in comparison to meat-based diets when vegan or vegetarian diets were fed [48,54,55,56,57,58,59,60,61,62,63] and only one study—with the smallest sample size and study conditions furthest removed from the normal environments of domesticated dogs—demonstrated a contrary result [64]. Three credible studies had assessed health outcomes in cats fed vegan or vegetarian diets, all of which similarly demonstrated equivalent or superior health outcomes [47,65,66]. Additionally, a systematic review examined the health impacts of vegan and vegetarian diets in dogs and cats, concluding that “there was no overwhelming evidence of adverse effects arising from use of these diets and there was some evidence of benefits” [14]. Across the studies reviewed, health outcomes were measured using a range of indicators, including radiographs (x-rays), blood nutrient levels, and biomarkers of organ health, veterinary clinical examinations, veterinary health assessments, clinical histories, guardian opinions of health, rates of medication use, and frequencies of veterinary visits.

This also makes sense biologically, when vegan pet diets are formulated to be nutritionally sound—as modern commercial diets normally are [67]—with concurrently lowered rates of certain dietary hazards, such as animal-sourced allergens, which can affect animals with allergies or dietary sensitivities. Additionally, by 2024, over eighty studies had demonstrated increased pathogenic hazards associated with raw meat diets [68]. Hence, animals maintained on nutritionally sound vegan diets can be expected to have health outcomes equivalent, or in some respects better, than those of pets fed meat-based diets. This is indeed what the collective studies to date have demonstrated.

Fewer studies exist so far demonstrating positive health outcomes in cats, compared to dogs. However, from an EA perspective, the primary focus should be on dogs in any case. Under a global transition to nutritionally sound vegan diets, the potential sustainability benefits of canine dietary change are around six to seven times greater than those that would accrue from feline dietary change [22], reflecting the larger global canine population compared to cats, and their higher intake of animal-sourced ingredients.

A recent study also assessed the safety of the microbial protein ‘FeedKind’ in 32 adult dogs fed diets containing between 0 to 8% inclusion of the protein, over a six-month period [69]. Blood, urine, and fecal samples were analyzed throughout the study, and body weight was monitored. The authors concluded that the inclusion of FeedKind protein “at up to 8% of the total diet of adult dogs can provide sufficient nutrition and is safe with no long-term effects on a range of health parameters”.

#### 4.2.2. Nutritional Soundness and Quality

The nutritional soundness and quality of plant-based vs. meat-based pet foods were recently studied [67]. Twenty-nine pet food manufacturers were surveyed, 19 of which produced meat-based diets, with 10 producing plant-based diets (vegan, vegetarian, or ‘almost vegan’ diets—those containing minimal animal-derived ingredients, such as vitamin D3 from lanolin found in sheep’s wool). The companies were predominantly based in the UK and other European countries, although their marketing regions also extended to Asia, Oceania, and North America. The results indicated that all 29 companies maintained acceptable or superior standards across nearly all stages of formulation, manufacturing, and distribution, with plant-based diets performing slightly better than meat-based diets overall. This might have occurred as the plant-based pet diets were newer and more controversial, and so companies might have been taking extra care to ensure nutritional soundness through appropriate nutritional supplementation and other steps [67]. Brociek et al. [70] also recently conducted a nutritional analysis of 31 commercially available complete dry dog foods in the UK and found that vegan and vegetarian diets provide nutrition comparable to their meat-based counterparts.

#### 4.2.3. Palatability

Current evidence suggests that palatability concerns (e.g., [71]) around sustainable diets are unfounded. A colleague and I previously surveyed 4060 dog or cat guardians who fed their companion animals either conventional meat-based, raw meat-based, or vegan diets [72]. Guardians were asked about the extent to which their animals displayed a wide range of species-specific palatability behaviors at mealtimes, e.g., lip licking, eating quickly, and guarding food. We found no consistent differences in palatability indicators between vegan diets and either conventional or raw meat-based diets.

#### 4.2.4. Availability

Product availability is another important factor affecting consumer uptake. The sustainable pet food sector has expanded rapidly in recent years, with vegan pet diets relatively widely available in many regions by 2026. A review by Klinmalai et al. [73] reported significant increases in product launches and patents for pet foods containing alternative proteins—including cultivated meat, and protein from terrestrial and aquatic plants. Between 2014 and 2024, 89 new pet foods marketed as plant-based were recorded in Mintel’s global new product database. Similarly, an analysis of 4563 dog foods sold in Europe (2020–2024) found that, relative to animal-based and hybrid (plant and meat combined) products, the fully plant-based product segment “experienced the most significant growth, particularly post-2020, with launches surging from nine in 2020 to 57 in 2024” [74]. Hybrid products showed the greatest overall increase in new launches, while the number of new animal-based products declined slightly. The authors concluded that these trends reflect rising consumer interest in ethical and sustainable pet food options. This momentum is expected to continue. As noted previously, the global vegan pet food market was valued at USD 27 billion in 2024 [10] and is projected to more than double in value to reach USD 57 billion by 2034. This represents a compound growth rate of 7.8% [11].

#### 4.2.5. Veterinary Approval

Veterinarians are increasingly aware of the growing body of evidence supporting the use of nutritionally sound vegan pet diets (see Section 4.2.1 ‘Health outcomes’). In July 2024, the British Veterinary Association (BVA)—the main UK veterinary professional association—ended its opposition to such diets for dogs, stating in its updated policy position that “it is possible to feed dogs a plant-based diet” [75]. The BVA did not attempt to determine the ‘best’ diet for companion animals but instead emphasized the importance of “supporting pet owners to ensure they are meeting their pets’ nutritional needs as well as meeting their own lifestyle choices.” As the benefits of sustainable pet diets continue to gain recognition among veterinary professionals, similar shifts in policy positions are expected from veterinary authorities in other countries.

## 5. Conclusions

Based on these factors, the production of conventional meat-based pet food is an issue that appears very well matched to the EA criteria of scale, neglectedness, and tractability. Globally, by 2018, around 9% of all farmed animals were fed to companion animals, rising to 20% in developed nations with high pet guardianship, such as the US [22]. If all companion dogs and cats transitioned to nutritionally sound vegan diets, approximately seven billion farmed land animals and many billions of marine animals could be spared annually. Furthermore, the food energy savings associated with this dietary shift could feed an additional 519 million people, or approximately 160% and 190% of the 2018 global populations of companion dogs and cats, respectively [22]. The environmental benefits are similarly profound; such a transition just for dogs could reduce GHG emissions by an amount greater than those produced annually by the UK, free up land areas larger than Mexico, and conserve freshwater volumes exceeding all renewable freshwater in Denmark [22].

Additionally, this issue appears highly tractable. By 2026, vegan pet diets were relatively widely available in many regions, often through online retailers. And two recent, large-scale surveys indicated that, among guardians currently feeding their companion animals meat-based diets, 18.2% of cat guardians and 13.4% of dog guardians would be open to transitioning their companion animals to vegan diets, if their concerns about these diets were addressed. Accordingly, assuming only one dog or cat per guardian, at least 70 million dogs and 86 million cats worldwide could potentially be transitioned to vegan diets. However given that many guardians actually have multiple pets, the true totals are probably several times higher. A greater proportion of dog and cat guardians would be open to cultivated meat-based pet diets—24.4% and 33.1%, respectively [45,46]—although these were not yet readily available in most regions by early 2026. Importantly, there is already a significant body of evidence addressing guardians’ key concerns about sustainable pet diets, which include the health outcomes, nutritional adequacy, and palatability achieved by these diets.

However, despite the significant potential benefits for pets, farmed animals, people, and the environment, efforts to increase consumer demand for sustainable pet food remain severely neglected. The total funding received for vegan pet food research and advocacy is estimated to be very far below USD 1 million annually, representing a great deal less than 0.5% of the farmed animal advocacy movement’s budget. Given its scale, tractability, and neglectedness, the issue of meat-based pet food warrants significantly more attention from the EA community and beyond. Effective altruists, animal advocates, and environmentalists should broaden their anthropocentric focus on human dietary change. 

Globally, by 2018, fewer farmed land animals were consumed within the diets of ordinary people (9) than average dogs (13) each year, and this disparity is increasing, as global dog populations grow faster than human populations. Shifts in companion animal diets provide a highly impactful, yet largely overlooked, opportunity to reduce farmed animal use and numbers slaughtered, improve global food security, and mitigate the climate and biodiversity crises. This field urgently warrants more resourcing—in terms of funding, time, and talent—to further strengthen the research base underpinning the development and use of nutritionally sound, sustainable pet diets, and to accelerate awareness and adoption of them worldwide.

## Figures and Tables

**Table 1:** Farmed land animals consumed in the US in 2020, to feed dogs, cats, and humans. After Knight [22].

	US Total (2020)	Humans (80.0%)	Per Human—Annually	Per Human—Lifetime	Dogs (17.7%)	Per Dog—Annually	Per Dog—Lifetime	Cats (2.3%)	Per Cat—Annually	Per Cat—Lifetime	Dogs and Cats (20.0%)
Poultry	9,592,147,000	7,673,717,600	23.32	1828.63	1,697,810,019	19.67	249.65	220,619,381	3.61	40.37	1,918,429,400
Pigs	131,639,000	105,311,200	0.32	25.10	23,300,103	0.27	3.43	3,027,697	0.05	0.55	26,327,800
Bovine animals	33,366,100	26,692,880	0.08	6.36	5,905,800	0.07	0.87	767,420	0.01	0.14	6,673,220
Sheep and goats	2,942,800	2,354,240	0.01	0.56	520,876	0.01	0.08	67,684	0.00	0.01	588,560
Other land animals	77,594	62,075	0.00	0.01	13,734	0.00	0.00	1785	0.00	0.00	15,519
**Totals**	**9,760,172,494**	**7,808,137,995**	**23.73**	**1860.66**	**1,727,550,531**	**20.02**	**254.03**	**224,483,967**	**3.67**	**41.08**	**1,952,034,499**

**Table 2:** Farmed land animals consumed globally in 2018, to feed dogs, cats, and humans. After Knight [22].

	World Total (2018)	Humans (91.1%)	Per Human—Annually	Per Human—Lifetime	Dogs (7.7%)	Per Dog—Annually	Per Dog—Lifetime	Cats (1.2%)	Per Cat—Annually	Per Cat—Lifetime	Dogs and Cats (8.9%)
Poultry	74,640,136,000	67,997,163,896	8.85	649.53	5,747,290,472	12.20	154.85	895,681,632	2.40	26.85	6,642,972,104
Pigs	1,478,059,606	1,346,512,301	0.18	12.86	113,810,590	0.24	3.07	17,736,715	0.05	0.53	131,547,305
Sheep and goats	1,047,391,220	954,173,401	0.12	9.11	80,649,124	0.17	2.17	12,568,695	0.03	0.38	93,217,819
Other land animals	726,797,375	662,112,409	0.09	6.32	55,963,398	0.12	1.51	8,721,569	0.02	0.26	64,684,966
Bovine animals	353,868,375	322,374,090	0.04	3.08	27,247,865	0.06	0.73	4,246,421	0.01	0.13	31,494,285
**Total**	**78,246,252,576**	**71,282,336,097**	**9.28**	**680.91**	**6,024,961,448**	**12.79**	**162.33**	**938,955,031**	**2.52**	**28.14**	**6,963,916,479**

## Data Availability

The original contributions presented in this study are included in the article. Further inquiries can be directed to the author.
